# Ductal Carcinoma In Situ of the Breast: An Update with Emphasis on Radiological and Morphological Features as Predictive Prognostic Factors

**DOI:** 10.3390/cancers12030609

**Published:** 2020-03-06

**Authors:** Lucia Salvatorelli, Lidia Puzzo, Giada Maria Vecchio, Rosario Caltabiano, Valentina Virzì, Gaetano Magro

**Affiliations:** 1 Department of Medical and Surgical Sciences and Advanced Technologies, G.F. Ingrassia, Azienda Ospedaliero-Universitaria “Policlinico Vittorio Emanuele”, Anatomic Pathology, School of Medicine, University of Catania, 95123 San Giovanni Galermo, Italy; lipuzzo@unict.it (L.P.); giadamariavecchio@gmail.com (G.M.V.); rosario.caltabiano@unict.it (R.C.); g.magro@unict.it (G.M.); 2 U.F. Radiodiagnostica Casa di cura Regina Pacis, 93017 San Cataldo, Italy; valentinavirzi@gmail.com

**Keywords:** DCIS, diagnosis, mammography, morphological features, immunohistochemical profile, prognosis

## Abstract

Ductal carcinoma in situ (DCIS) shows overlapping epidemiology with invasive ductal carcinoma of the breast, sharing similar risk factorssuch as age, mammographic density, family history, and hormonal therapy as well as genetic factors such as BRCA1/BRCA2, histotypes, and molecular subtypes such as luminal A and B, HER2 enriched, and basal-type, thus suggesting its potential precursor role. A small percentage of patients with a history of DCIS die without a documented intermediate diagnosis of invasive breast carcinoma (IBC). The increased risk of death is usually associated with ipsilateral recurrence such as IBC. The slightly variable incidence of DCIS in different countries is mainly due to a different diffusion of mammographic screening and variability of the risk factors. The majority of DCIS lesions are not palpable lesions, which can be only radiologically detected because of the association with microcalcifications. Mammography is a highly sensitive diagnostic procedure for detecting DCIS with microcalcifications, while magnetic resonance imaging (MRI) is considered more sensitive to detect DCIS without calcifications and/or multifocal lesions. The aim of the present overview was to focus on the clinical, radiological, and pathological features of DCIS of the breast, with an emphasis on the practical diagnostic approach, predictive prognostic factors, and therapeutic options.

## 1. Definition

Ductal carcinoma in situ (DCIS) is a non-obligate precursor of invasive breast carcinoma. The great interest for this pre-invasive lesion lies in the fact that its early diagnosis and appropriate treatment are crucial to prevent the development of an invasive cancer that can be potentially lethal. DCIS is a segmental disease arising from a terminal duct lobular unit with the potential to progress within the duct system up to the lactiferous ducts and nipple. DCIS is a malignant proliferation of ductal epithelial cells that grow with different endoluminal architectural patterns, but restricted to the ductal-lobular system, and thus without documented stromal invasion. This means that either the basal membrane or the layer of myoepithelial cells isstill preserved, preventing the possibility of the neoplastic cells to metastasize. Although a unifying term, DCIS is used inthis highly heterogenous diseasein terms of extension, morphology, biology, and prognosis [1].

## 2. Epidemiology

It is widely accepted that most cases of DCIS are now diagnosed through breast cancer screening programs and 20% of all breast carcinomas are “in situ” lesions [2]. As most DCIS are surgically treated, it is not surprising that approximately 20–25% of surgical breast samples evaluated by pathologists refer to this disease [3,4]. The incidence of DCIS has increased over the last three decades, ranging from 1.87 cases per 100,000 person-years in the first years of the 1970s to 32.5 cases per 100,000 person-years in 2005 [5]. Breast carcinoma is the most frequent cancer among women in different age groups, with 40% of cases diagnosed in the 0–49 age group, 35% of cases in the 50–69 age group, and 22% of cases in the oldest age group >70 years. It has been calculated that one case of DCIS is usually identified per 1000 screening mammograms.

## 3. Clinical Features

Most DCIS lesions are diagnosed mammographically (70–90%), being rarely detected clinically. Only a few cases of DCIS (2–3%) present as a small-sized (≤1 cm) palpable mass, or with nipple changes (discharge or Paget’s disease). Notably a small percentage (up 5%) of DCIS lesions is incidentally detected at the histological examination of breast tissue evaluated for other reasons.

## 4. Radiologic Features

Most DCIS lesions are not usually palpable lesions, being only radiologically detected. Mammography is a highly sensitive diagnostic procedure for detecting DCIS. Several studies show that the diagnosis of DCIS mainly depends on the detection of microcalcifications on mammographic screening (70–90% of cases) [4,6,7], while only a smaller percentage of cases is diagnosed as a palpable mass or a nipple discharge or ulceration (Paget’s disease). In 2002, it was estimated that about 1/1300 screening mammographies was consistent with the diagnosis of DCIS, histologically proven by needle core biopsy [8]. Microcalcifications alone are likely the most reliable mammographic indicators of DCIS in women younger than 50 years, whereas parenchymal abnormalities, especially architectural distortions, are the alarming features more evident in women older than 50 years, due to the variation in overall breast density at this age [9]. Briefly, two types of microcalcifications are recognized: fine and coarse microcalcifications, with a clustered (at least five microcalcifications in a small volume of tissue: <1 cc) or linear distribution (ductal extension); round and/or oval, well-circumscribed calcifications are described less commonly [9]. In addition, calcifications may be clustered, dispersed, or dispersed around clustered foci. Branching calcifications with linear patterns outlining the ductal distribution may consist of casts or granular/round particles.

**Figure 1:**
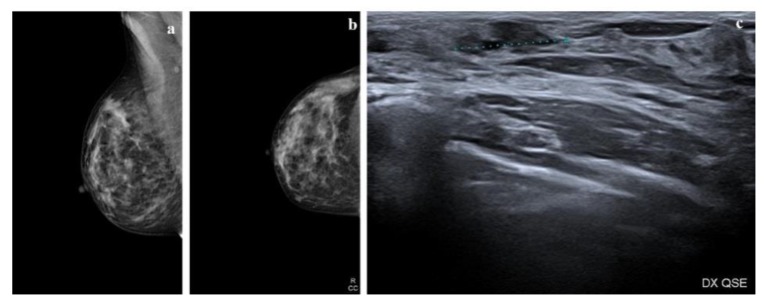
Radiologic features of a low-grade ductal carcinoma in situ (DCIS) lesion. (**a**,**b**) Mammograms show a heterogeneously dense right breast. In the upper outer quadrant (UOQ) there is a small oval opacity with obscured margins; (**c**) ultrasound shows an irregular mass with indistinct margins, hypoechoic echo pattern without posterior features.

**Figure 2:**
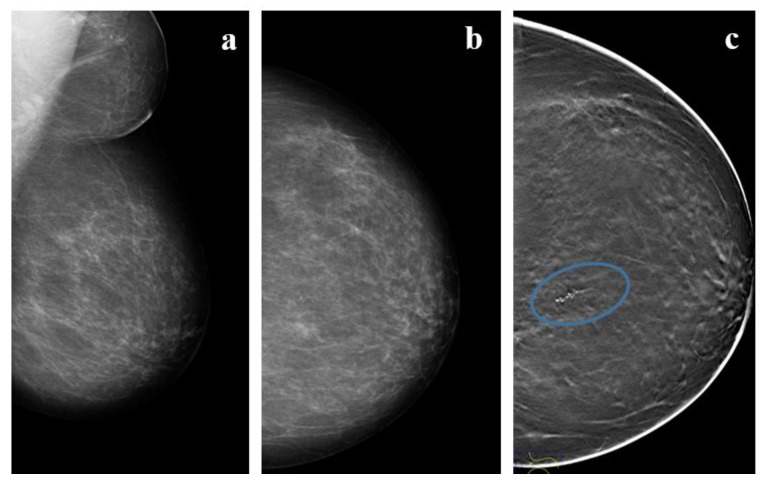
A 57 year-old woman with high-grade DCIS. (**a**,**b**) Mammograms and (**c**) Tomosyinthesis imaging of the left, almost entirely fatty, breast shows a cluster of fine-linear branching calcifications (circle) in the upper inner quadrant (UIQ) classified using breast imaging-reporting and data system (BI-RADS) as Category IVc.

**Figure 3:**
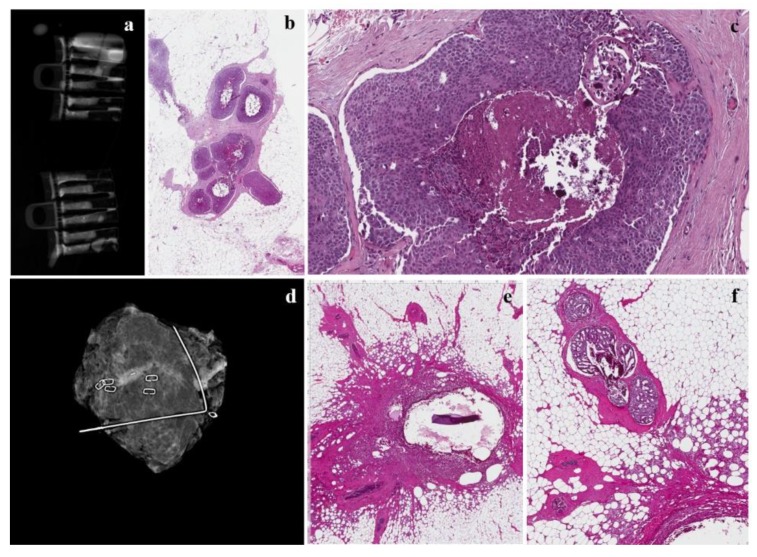
The same case as Figure 3. (**a**) Radiograph during localization of the microcalcification in the samples collected in touch-free collection chambers using the Mammotome Revolve 10 gauge biopsy system that reveals numerous microcalcifications in the cores; (**b**,**c**) mammotome biopsy shows a high-grade DCIS with central comedonecrosis at low- and high-magnification; (**d**) mammogram revealing successful retrieval of a cluster of pleomorphic calcifications with prior localization and subsequent surgery; (**e**) inflammatory reaction around the previously placed clips; (**f**) a residual focus of cribriform carcinoma in situwith central microcalcifications.

Although the type of microcalcifications is unrelated to the histological subtype (solid, papillary, cribriform), there is a relatively good correlation with tumor grade: multiple clusters of fine granular calcifications are usually associated with low-grade lesions (LG-DCIS) (Figure 1), while linear, often branching, or coarse granular microcalcifications are commonly found in high-grade lesions (HG-DCIS) (Figure 2 and Figure 3). Mammographic distribution of microcalcifications is commonly used as a guide to assess DCIS size or the dimension of the involved area. As the distribution of microcalcifications may be heterogeneous, including missing areas, it should be borne in mind that the real extension of DCIS lesions could be underestimated by mammography [10]. Tomosynthesis, a new three-dimensional (3D) mammographic imaging technique, is emerging as a new screening tool for improved breast cancer detection, mostly in invasive cancer (Figure 1 and Figure 3). A recent population-based study found that 1 out of 16 cases of DCIS was detected only by tomosynthesis, compared with 28 out of 74 invasive breast cancers.

## 5. Magnetic Resonance Imaging

Magnetic resonance imaging (MRI) has been considered more sensitive than mammography for detecting DCIS without calcifications and multifocal lesions [11]. However, several authors report that MRI shows higher sensitivity for invasive carcinoma (up to 98%) than for DCIS (60–80%): DCIS is typically not mass-forming, with adelayed peak enhancement profile; MRI can miss low-grade DCIS, while it is more sensitive for high-grade DCIS, showing higher vascularity. A comparison of morphological and immunohistochemical studies with MRI features of DCIS suggests that tumor angiogenesis could contribute to MRI enhancement [12,13].

Contrast-enhanced MRI has been considered an effective method for the detection of a concurrent contralateral carcinoma in situin women with a previous diagnosis of ipsilateral DCIS, an occult primary tumor, an examination of dense breast tissue, a study of breasts in patients with BRCA mutations, and neoplastic involvement of the chest wall.In the evaluation of DCIS, MRI could be useful to assess disease extent, showing a diagnostic accuracy of more than 60%, compared tomammographic accuracy of 55%; however, this methodology has a high rate of false negatives.

## 6. Morphological Features of Carcinoma In Situ

**Figure 4:**
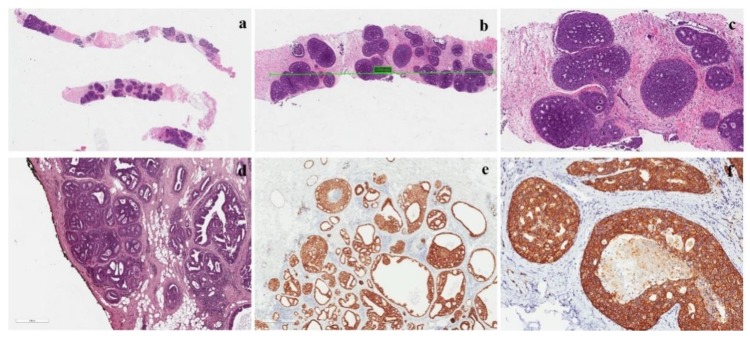
Features of a low-grade DCIS. (**a**) Low-grade DCIS in a core needle biopsy; (**b**,**c**) intermediate and high-magnification of the lesion showing a cribriform growth pattern; (**d**) DCIS with negative margins, but the distance between neoplasia and inked margin is <2 mm; (**e**) ER positivity in a low-grade DCIS; Her-2 positivity (score 3+) in a low-grade DCIS.

DCIS is a unifocal disease originally restricted to a single duct system but with the capability to involve different lobules. The basal membrane and the layer of myoepithelial cells are—by definition—preserved. The latter cells can be easily highlighted by means of immunohistochemistry using different immunomarkers such as p63, high molecular-weight keratins 5/6, 14, calponin, S100, and smooth muscle actin. DCIS is a heterogeneous disease not only biologically and genetically but also morphologically. The pathology report should include several features according to the College of American Protocol of Pathologists [14]: nuclear grade, architectural growth pattern, necrosis, stromal reaction, tumor-infiltrating lymphocytes, immunohistochemical profile (estrogen receptor-ER, Progesteron receptor-PgR, HER-2), size and extension, and surgical margins. Based on nuclear grade atypia, DCIS can be categorized as low, intermediate, and high nuclear grade lesions. It is not unusual to detect two different nuclear grades in the same lesion as the result of genetic heterogeneity. Low-grade DCIS is a proliferation of small and monomorphic neoplastic cells, with inconspicuous nucleoli and few mitoses (Figure 4). Conversely, high-grade DCIS shows large-sized, pleomorphic neoplastic cells with large and irregular nuclei, multiple and prominent nucleoli, high mitotic index, and often necrosis (Figure 3). Intermediate-grade DCIS is a neoplasia with overlapping features between low- and high-grade DCIS. Numerous meta-analysis studies suggest that high-grade DCIS shows an increased risk of ipsilateral recurrence compared to low-grade DCIS. However, Zhang et al. in his meta-analysis shows that high- and intermediate-grade DCIS are not significantly associated with the risk of local invasive recurrence [15]. DCIS may exhibit different growth patterns, including (i) cribriform, in which neoplastic cells form round and rigid arches giving a holing aspect to the luminal space (Figure 4); (ii) papillary, in which neoplastic cells show a papillary architecture with a fibrovascular core; (iii) micropapillary, in which tufts of monomorphic neoplastic cells without a fibrovascular core, protrude into the glandular lumen; and (iv) solid, in which neoplastic cells form sheets filling the glandular lumen. Several studies correlate solid, papillary, and micropapillary growth patterns with a more consistent and strong adverse outcome. High-grade DCIS often shows comedonecrosis, when the necrotic core extends into the ductal lumen that canundergo calcifications. Necrosis is also constantly and strongly associated with ipsilateral recurrence, with the hazard ratio (HR), in general, above 2.0 [16]. Some studies demonstrated that the risk of ipsilateral recurrence is greater if DCIS with comedonecrosis is only treated with breast conserving surgery, compared to DCIS managed with mastectomy, skin-sparing mastectomy, or breast conserving surgery plus radiotherapy [16]. Zhang et al. also disagreeon the role of comedonecrosis, suggesting that there is no correlation between necrosis and the risk of invasive local recurrence [15].

Reporting stromal reaction and tumor-infiltrating lymphocytes (TILs) is also recommended. High-grade DCIS is predominantly associated with a desmoplastic reaction of the periductal stroma and seems to correlate with a higher risk of recurrence [17]. TIL is a dense, chronic inflammatory infiltrate surrounding DCIS. Several studies demonstrated that breast carcinomas with a marked intra-tumoral stromal lymphocytic infiltrate have a better prognosis than carcinomas with lymphocyte depletion [18,19]. Triple-negative and HER2-positive DCIS lesions are the subgroups of breast carcinomas that show the greatest degree of enrichment of the stroma by lymphocytes (tumor infiltrating lymphocytes, TILs) [20]. A shorter recurrence-free interval is seen if TILs are associated with younger age, larger size, comedonecrosis and ER negative, and HER2 overexpression. However, no significant association was identified for longer follow-ups [21]. It should be noted that the evaluation of TILs has a prognostic value and not a predictive value of response to therapies, therefore, this criterion is not used to decide whether or not to administer chemotherapy or other systemic therapies.

DCIS tumor size could be difficult to measure on multiple slides; DCIS with a ≤20 mm diameter is usually considered “small”. Several meta-analyses showed that among tumor characteristics, tumor size was certainly positively correlated with a higher rate of recurrence of ipsilateral breast cancer, although many of the estimates were not statistically significant [22]. Conversely, in another meta-analysis, Zhang et al. disprove that tumor size is significantly associated with the risk of local invasive recurrence [15]. DCIS multifocality is defined as multifocal foci with at least 5 mm of intervening healthy tissue confined to a single breast quadrant [23]. Several studies of meta-analyses correlate multifocal DCIS with increased risk of recurrence with an estimated risk of 1.95 [22]. Conversely, Zhang et al. show a multifocal DCIS lesion is also associated with an increased risk of recurrence for invasive ductal carcinoma [15].

Several predictive score systems have been developed for DCIS, mainly based on pathological features. A recent clinical risk score, proposed by Punglia et al., seems to be useful and easy to use [24]. This score system includes a large number of patients (*n*. 2762) treated with breast-conserving surgery (BCS) alone or plus radiation and/or hormonal therapy. The following factors were found to be associated with ipsilateral recurrence within 5 years: (i) age ≤50 years; (ii) comedonecrosis; and (iii) ER-negative status. It was calculated that the 5-year risk of ipsilateral recurrence after BCS alone was 9% for the low-risk group, 23% for the intermediate-risk group, and 51% for the high-risk group. Based on these findings, clinicians, after establishing the patient’s risk-group, can guide subsequent adjuvant treatment (radiation with or without hormonal therapy) for patients with negative margins after BCS.

## 7. Immunohistochemical Profile

Although several immunohistochemical studies have been performed on DCIS, with the aim of correlating a specific immunohistochemical profile with the risk of local recurrence (in terms of in situor invasive lesions), the results are conflicting [16]. This is true not only for each single immunomarker tested but also after classifying DCIS into molecular subtypes, similarly to what has been applied to invasive breast carcinoma [25]. Although ER-status has been incorporated as one of the major pathologic factors, along with comedonecrosis, in a clinical score system for predicting DCIS recurrence [24], the results of two clinical studies, UKANZ and NSABP B-24, suggest that ER status is a weakly prognostic biomarker for local recurrence, but it is a strong predictor of response to endocrine therapy to reduce local recurrence. Currently, ER is the only useful marker for planning a potential endocrine therapy, and it should be performed on excision rather than in core biopsy.

## 8. Molecular Features

Breast cancer does not yet have a molecular signature that can predict the risk of recurrence or that may indicate treatment. To date, the only validated and available molecular marker is Oncotype DX Breast DCIS Score, which evaluates 12 genes derived from the 21 genes used for calculating the Oncotype DX Recurrence Score for early invasive breast cancer. The DCIS score can be used to quantify the risk within 10 years of developing a relapse, even invasive, following the diagnosis of DCIS without radiotherapy. The histopathological aspects that correlate with the score are unclear, but several studies explain that the predictive value of this score assumes greater value if correlated with age at diagnosis, tumor size, and multifocality [16].

## 9. Surgical Margins

The standard treatment of DCIS is primarily surgical, including BCS for localized lesions, and mastectomy for extensive or multicentric disease. Local recurrence rates (LRs) after BCS alone are high, ranging from 25% to 35% at 13–17 years of follow-up, and approximately half of all recurrences are invasive [26]. As most patients with negative margins after BCS are at lower risk of LR, the optimal margin width has been a matter of debate for many decades. Although multiple studies have shown that positive margins or close to margins are both associated with a higher risk of LRs, there is no consensus about the optimal adequate margin width. This is also complicated by the fact that many patients treated by surgery, usually undergo subsequent whole breast radiation therapy (WBRT). The Society of Surgical Oncology (SSO), the American Society for Radiation Oncology (ASTRO), and the American Society of Clinical Oncology (ASCO) concluded that a positive margin, defined as ink on tumor (DCIS), is associated with an increased risk in LR and that such risk is not nullified by the use of WBRT. They also concluded that the free margin for BCS with WBRT is 2 mm [27]. At the same time the LORIS trial metanalysis [28] of patients with DCIS, undergoing BCS plus WBRT, concluded that margin distances above 2 mm are not significantly associated with a further reduction in odds of LR. Even if histologic grade and architectural patterns, comedonecrosis, tumor size, and gene expression profiles are associated with the risk of LR, they did not influence the recommended margin width. The European Society for Medical Oncology (ESMO), the National Institute for Health and Clinical Excellence (NICE), and the New Zealand Guidelines Group established that an appropriate margin width should be 2 mm [29,30,31]. The National Comprehensive Cancer Network guidelines now states that margins of at least 2 mm are associated with a reduced risk of ipsilateral breast tumor recurrence [32]. A different perspective comes from the American Society of Breast Surgeons that considers exclusively *“no ink on the tumor”* as a negative margin [33]. Notably the MD Anderson experience [34], corroborated by the Memorial Sloan Kettering Cancer Center [35], reported that the difference in Local recurrence rate (LRR) for patients with margins <2 mm vs. ≥2 mm (10-year LRR 30.9% vs. 5.4%, respectively; *p* = 0.003) was abrogated in patients receiving radiotherapy (10-year LRR 4.8% vs. 3.3%, respectively; *p* = 0.72). As many authors state that the strongest predictor of local recurrence of DCIS after BCS is a positive surgical margin at the time of initial surgery, a careful macroscopic sampling and microscopic examination must be carried out. If DCIS has an adequate distance (neoplasia-negative margin: ≥2 mm), no re-excision is required. Conversely, if the negative margins are ≤2 mm, other parameters such as age, tumor extension, and nuclear grade, must be evaluated before performing surgical re-excision. Recent studies have demonstrated that the risk of recurrence is similar between patients who underwent radiotherapy, both with 0–1 mm or ≥2 mm margins.

## 10. Sentinel Node Sampling

DCIS is—by definition—a carcinoma without invasion of the basement membrane and thus the examination of axillary nodes is indicated only for selected patients. Indeed, the occurrence of lymph node metastases from ductal carcinoma in situ is possible. The pathogenesis may be due to a missing area of microinvasion from a large-sized DCIS or iatrogenic dissemination of tumor cells evaluated by the preoperative breast biopsy. A multi-institutional audit performed at the Memorial Sloan-Kettering center reported a 9% incidence (43/470) of positive sentinel nodes after DCIS [36]. The American Society of Clinical Oncology Clinical Practice Guideline recommends sentinel lymph node biopsy (SLNB) for women who undergo mastectomy, or with high-grade and large-sized tumors (>5 cm), or in the presence of multicentric disease or tumor mass diagnosed by an imaging study suspicious for invasive cancer. The reason to perform SLNB for patients undergoing mastectomy is the impossibility to perform this diagnostic procedure in the event that an invasive cancer is detected. Similarly, SLNB should be performed if the lumpectomy will compromise drainage enough to prevent a future sentinel lymph node procedure. This is most often considered for DCIS lesions located in the high axillary tail.

## 11. Natural History

It is extremely difficult to trace the natural history of DCIS. As in the last decades the rate of invasive recurrence of DCIS has not changed significatively, despite an increased rate of diagnoses basedon mammographic screening, it is likely that the majority of patients with DCIS does notdevelop invasive carcinoma. This is why DCIS should be regarded as a “non-obligate” precursor of invasive carcinoma. This concept is in line with what we know from carcinomas in situ occurring in other organs. We can speculate about the natural history of DCIS by evaluating the clinco-pathological studies based on the recurrence rate after a histologicallyproven diagnosis on core biopsy. A long-term follow-up study, based on a systemic review, meta-analysis, and meta-regression analysis, revealed a local recurrence rate of 40% after 15years [37]. Notably only 28.1% of the recurrence was in the form of invasive carcinoma associated with a mortality rate of 18%**.** The most updated view about DCIS considers this disease as a wide morphological and biological spectrum, ranging from small-sized, usually low-grade, lesions that can be treated by surgical excision alone, to extensive, often high-grade lesions, for which the best treatment seems to be mastectomy. The largest studies on the natural history of DCIS suggest that more than 50% of patients with high-grade DCIS have the potential to progress to an invasive carcinoma in less than 5 years if left untreated, while low-grade DCIS has a similar progression but in a small percentage of patients (35–50%) and in a more prolongated time course, up to 40 years [38,39,40,41].

## 12. Treatment

Based on the notions from the natural history of DCIS, patients with a histologically proven diagnosis should be treated to prevent the possibility of local recurrence both in terms of non-invasive or invasive carcinoma. DCIS treatment is still controversial, with a wide possibility of options, including surveillance, breast-conserving surgery (BCS), BCS in association with radiotherapy and/or hormonal therapy, and mastectomy with or without radiotherapy. Over time, there have been many efforts to identify patients with low- or high-risk DCIS lesions, in order to avoid, respectively, over- or under-treatment. The option of an active surveillance could be offered not only to older patients, but also to all patients with mortality risks due to other diseases [42]. As far as surgery is concerned, mastectomy, with immediate or subsequent breast reconstruction, is strongly recommended for those patients with large-sized tumors, multifocal tumors, small-sized breasts (cosmetic problems), family history, or documented BRCA mutations. Conversely, the proposal of BCS alone or in combination with radiotherapy is still a matter of debate. Although BCS alone seems to be an effective treatment, it is not enough for DCIS. This is supported by the evidence that patients both with low/intermediate-grade and high-grade DCIS (<10 mm and with negative margins: ≥3 mm) treated with BCS alone, experienced ipsilateral recurrence (both in situ and invasive disease) at a 12-year of follow-up, respectively, in 14% and 25% of cases [43]. In addition, several studies revealed that adjuvant radiotherapy has a great impact in reducing by ≈50% the risk of ipsilateral recurrence in terms of invasive carcinoma at 10 and 15 years follow-up, compared with surgery alone [30,44,45,46]. Studies with longer follow-up periods have also shown that radiotherapy is capable of decreasing the risk of ipsilateral recurrence by 10% and 2%, respectively, as DCSI or invasive carcinoma at 20 years [47]. Hormonal therapy is an effective adjuvant treatment in reducing both ipsilateral and contralateral events by ≈30% at 10 and 15 years of follow-up in ER-positive DCIS [48,49]. Tamoxifen may be more efficacious in patients receiving concurrent adjuvant radiotherapy. Aromatase inhibitors, such as anastrozole, seem to have an age-dependent superiority to tamoxifen, which is more effective in older patients (>60 years) [49].
